# 

**Published:** 1885-06-20

**Authors:** 


### TABLE OF CONTENTS.

ORIGINAL AND SELECTED ARTICLES:

Convulsions in Children, by Arch. Dixon, M.D., of Kentucky, 201. Kairine and Antipyrine in the Treatment of Fever, by P. R. Cortelyou, M.D., of Marietta, Ga,, 266. Cocaine in "Earache," by A. G. Hobbs, M.D., Prof. Eye, Ear, etc., Southern Medical Colllege, Atlanta. Ga., 209. Treatment of a Case of Cerebro-Spinal Meningitis, by J. McF. Gaston, M D.. Prof. Prin and Prac. Surg. Southern Med. College, Atlanta, Ga. "Colds," by George G. Groff, M.D., Lewisburg, Penn., 211. The Hypodermic Syringe, by J. S. Giegley, M. D., Lewiston, Ill., 214. Piles and Prolapsus Ani, by W. Symington Brown, M.D., Stoneham, Mass., 216. Treatment of Intussusception, by Frederick Treves, F.R.C.S., 219. Hints for Avoiding Rheumatism; Carbolic Acid in Indigestion.

ABSTRACTS AND GLEANINGS:

Obstetric Forceps, 221. Vinegar in Post-Partum Hemorrhage, 222. Hair Tonic, 223. Lobelia Inflata; Vacination during Incubation of Variola, 224. New Specific for Typhoid Fever, 225. Monsel's Iron in Diarrohœa; Can Tuberculosis be Transmitted by Vaccination, 226. Officinal Aqua Creasoti; Treatment of the Umbilical Cord, 227. Treatment of Fracture of the Clavicle; Carbolized Resin in In-growing Toe-nail, 228. Pilocarpia in Diptheria; Glycerin for Dryness of Tongue and Thirst in Febrile State; Influence of Diet on Headache; Antidote for Idoform, 229. Antipyretic Formula; Oranges for Deficient Milk Secretion; Disadvantages of Glycerin as a Solvent, 230.

SCIENTIFIC ITEMS:

About Snakes; Mercury Volatile, 231. Doctors Disagree; Case of Caudal Development in a Human Being, 232.

PRACTICAL NOTES AND FORMULÆ.

Dysentery; Dysentery; An Opthalmic Antiseptic Solution; A Diruetic Mixture, 233. Oleate of Bismuth in Eczema; Black Draught of British Pharmacopea an Excellent Purgative; Cough Remedy, 234.

EDITORIALS AND MISCELLANEOUS.

Editorial Notices; Late Action of the Georgia Medical Association Touching the Publication of its Actions, and What Others Think of it, 235. Books and Pamphlets Received, 239. Receits; Special Notices, 240.

